# Possible implication of vagal nerve stimulation for treating refractory psoriasis: Possível implicação da estimulação do nervo vagal para o tratamento da psoríase refratária

**DOI:** 10.1590/1516-3180.2015.00301103

**Published:** 2015-08-03

**Authors:** Nima Derakhshan, Mahboubeh Kazemi

**Affiliations:** I MD. Resident in Neurosurgery, Neuroscience Research Center, Shiraz University of Medical Sciences, Shiraz, Iran.; II MD. Obstetrician and Gynecologist, Department of Gynecology and Obstetrics, Shiraz University of Medical Sciences, Shiraz, Iran.

Psoriasis is a chronic, relapsing, immune-based skin disease that affects an estimated 1-3% of the world’s population.^1^ There is no consensus regarding the exact etiology of psoriasis; among several postulated pathophysiological causes, excessive activity of T cell-mediated immune response, and T-helper (Th) in particular, is the most accepted theory. This is supported by increased levels of pro-inflammatory cytokines, especially tumor necrosis factor (TNF-α) in the serum, skin lesions and joints of these patients.^2^^,^^3^

Although inhibitors of tumor necrosis factor (TNF-α) have been shown to be effective for treating refractory psoriasis,^3^^,^^4^ there are no therapies providing long-lasting remission for these patients.

Since Tracey^5^ proposed the so-called inflammatory reflex, the data now emerging has been elucidating and supporting the existence of a neural circuit that modulates immune response. This cholinergic anti-inflammatory pathway originates from efferent vagal fibers. It seems that inhibition of TNF-α production in the spleen following vagal nerve stimulation occurs through acetylcholine signaling via the α7 nicotinic acetylcholine receptor that is expressed on cytokine-producing macrophages.^5^^,^^6^ This signal is relayed through an acetylcholine-producing, memory phenotype T cell population that has been identified in mice, which is necessary for inhibition of cytokine production through vagus nerve stimulation.^7^

Immunomodulation via vagal nerve stimulation has been implicated in the treatment of other immune disorders involving the TNF-α pathway, such as inflammatory bowel disease.^8^ In this regard, we support the hypothesis that vagal nerve stimulation may prove useful for treating refractory psoriasis and psoriatic arthritis through its cholinergic anti-inflammatory effects, by means of modulating TNF-α production.^9^

This novel idea encourages interest in conducting a double-blind case-control study to investigate the possible role of vagal nerve stimulation in treating psoriasis and psoriatic arthritis.
